# Necrotizing Fasciitis

**DOI:** 10.21980/J84M1D

**Published:** 2020-04-15

**Authors:** Rishan Desta, Jennifer Yee

**Affiliations:** * The Ohio State University, Department of Emergency Medicine, Columbus OH

## Abstract

**Audience:**

This scenario was developed to educate emergency medicine residents and medical students on the diagnosis and management of necrotizing fasciitis (NF).

**Introduction:**

Necrotizing fasciitis is an uncommon and life-threatening deep tissue infection. Clinical presentations may range from subtle non-specific signs and symptoms to multi-organ failure.^1^ Signs and symptoms suggestive of necrotizing fasciitis include skin necrosis, hemorrhagic bullae, pain out of proportion, and erythema progressing beyond margins.^2^ Radiographs may show dissecting gas along fascial planes in the absence of trauma, but this is not a sensitive finding and may present late in the disease course. Ultrasound may demonstrate soft-tissue gas, fascial irregularity, and diffuse fascial thickening. Computerized tomography (CT) is the imaging modality of choice. The sensitivity of diagnosing necrotizing infections on CT is 80% and the diagnosis should not be excluded by lack of soft tissue gas.^3^ While laboratory findings may reveal an elevated C-reactive protein (CRP) level,^4^ other laboratory findings may be used in conjunction to calculate a laboratory risk indicator for necrotizing soft tissue infections score (LRINEC) value which may predict morbidity and mortality.^5^ Providers must treat empirically without delay or confirmation through laboratory results or imaging studies. Treatment should be initiated in a timely fashion, including broad-spectrum antibiotics, intravenous fluids, and urgent discussion with surgical consultants to evaluate for prompt operative management.^6^

**Educational Objectives:**

At the conclusion of the simulation session, learners will be able to: 1) Describe the spectrum of clinical presentations of necrotizing fasciitis. 2) Identify the microbial etiology of necrotizing fasciitis. 3) Describe the empiric antibiotics appropriate for necrotizing fasciitis. 4) Describe benefits and limitations of various imaging studies when working up necrotizing fasciitis.

**Educational Methods:**

This session was conducted using high-fidelity simulation, followed by a debriefing session reviewing case progression, differential diagnoses, management, and disposition. This scenario may also be run as an oral boards case.

**Research Methods:**

Our residents are provided a survey at the completion of the debriefing session so they may rate different aspects of the simulation, as well as provide qualitative feedback on the scenario.

**Results:**

Residents were also asked to rate the overall clinical experience of the simulation activity on a scale of 1 to 7; 1 being extremely ineffective/detrimental and 7 being extremely effective/outstanding. A total of 10 resident participants responded to the survey. This simulation scenario had a median rating of 7 for the instructor setting the stage and for instructors introducing themselves and explaining the learning objectives. A median rating of 7 was also given in explaining the strength and weakness of the simulation scenario, which was key to its clinical application.

Residents voiced appreciation that the clinical presentation was unique and that it required a thorough physical exam, including visualizing the affected area. As the patient presented rather early in his clinical course, they felt the diagnosis was more difficult to ascertain, as rhabdomyolysis and cellulitis were also on their differentials.

**Discussion:**

This is a cost-effective method to review a presentation of necrotizing fasciitis early in the disease course. Since the patient did not have a high fever or toxic appearance, participants demonstrated some hesitation in definitively diagnosing a necrotizing infection. We will continue to provide simulation scenarios with more subtle or early presentations to challenge residents’ diagnostic skills.

**Topics:**

Medical simulation, soft tissue infection, necrotizing fasciitis, infectious disease.

## USER GUIDE

[Table t1-jetem-5-2-s1]

**Table t1-jetem-5-2-s1:** 

List of Resources:	
Abstract	1
User Guide	3
Instructor Materials	5
Operator Materials	16
Debriefing and Evaluation Pearls	19
Simulation Assessment	21

**Learner Audience:**

Medical students, interns, junior residents, senior residents

**Time Required for Implementation:**

Instructor Preparation: 30 minutes

Time for case: 20 minutes

Time for debriefing: 40 minutes

**Recommended Number of Learners per Instructor:**

3–4

**Topics:**

Medical simulation, soft tissue infection, necrotizing fasciitis, infectious disease.

**Objectives:**

By the end of this simulation session, the learner will be able to:

1. Describe the spectrum of clinical presentations of necrotizing fasciitis
2. Identify the microbial etiology of necrotizing fasciitis
3. Describe the empiric antibiotics appropriate for necrotizing fasciitis
4. Describe benefits and limitations of various imaging studies when working up necrotizing fasciitis

### Linked objectives and methods

This simulation scenario will highlight the varied clinical presentations of NF (objective 1), which is dependent on the infection’s time course, underlying medical conditions, and causative organism(s) (objective 2). Taking these variables into consideration, broad-spectrum antibiotics should be administered early, including clindamycin to take advantage of its anti-toxin effects against staphylococcus and streptococcus strains (objective 3). The benefits and potential pitfalls of plain films and CT scans in NF should be weighed (objective 4) without delaying definitive operative debridement.

### Recommended pre-reading for instructor

We recommend that instructors review literature regarding necrotizing fasciitis, including its diagnosis and management. Suggested reading may be found under “References/suggestions for further reading.”

### Results and tips for successful implementation

This simulation was written to be performed as a high-fidelity simulation scenario, but also may be used as a mock oral board case. The case was written for emergency medicine residents and may be presented in a freestanding, community-based, or academic emergency department setting. We piloted a necrotizing fasciitis simulation case for approximately 20 emergency medicine residents during the 2018–2019 academic year. Participant feedback was overall positive. Participants voiced appreciation of the challenging quality of the case in that the patient presented early in the clinical course, the presentation of the affected area was subtle, and learners had to appropriately expose the patient to visualize the affected skin. They also felt they were able to keep a reasonably high index of suspicion for NF in this patient even though rhabdomyolysis and cellulitis were also part of their differentials. A picture of necrotizing fasciitis was included in the stimulus inventory which may be used to help guide moulage, but also may be cropped and shown to learners as a visual stimulus.

## Supplementary Information



## Figures and Tables

**Table t2-jetem-5-2-s1:** 

#1	Complete blood count (CBC)
#2	Basic metabolic panel (BMP)
#3	Liver function panel
#4	Lactic acid
#5	Creatine kinase (CK)
#6	Venous blood gas (VBG)
#7	Urinalysis
#8	Electrocardiogram (ECG)
#9	Chest Radiograph (CXR)
#10	Computed tomography (CT) right lower extremity
#11	External skin findings

**Table t3-jetem-5-2-s1:** 

**Vitals:**	Heart Rate (HR) 126	Blood Pressure (BP) 110/68	Respiratory Rate (RR) 26
	Temperature (T) 100.3°F	Oxygen Saturation (O_2_Sat) 98% on room air	
	Weight (Wt) 100 kg		

**Table t4-jetem-5-2-s1:** 

*Complete blood count (CBC)*	
White blood count (WBC)	21.0 ×1000/mm^3^
Hemoglobin (Hgb)	11.0 g/dL
Hematocrit (HCT)	33.0%
Platelet (Plt)	450 ×1000/mm^3^
Segs:	70%
Bands:	15%

**Table t5-jetem-5-2-s1:** 

*Basic metabolic panel (BMP)*	
Sodium	138 mEq/L
Chloride	100 mEq/L
Potassium	4.2 mEq/L
Bicarbonate (HCO_3_)	12 mEq/L
Blood Urea Nitrogen (BUN)	60 mg/dL
Creatine (Cr)	2.2 mg/dL
Glucose	85 mg/dL
Calcium	8.0 mg/dL

**Table t6-jetem-5-2-s1:** 

*Liver Function Test (LFT)*	
Total bilirubin	0.8 mg/dL
Direct bilirubin	0.2 mg/dL
Albumin	3.0 g/dL
Alkaline Phosphate	100 U/L
Total Protein	7.0 g/dL
Aspartate Aminotransferase (AST)	30 U/L
Alanine Aminotransferase (ALT)	40 U/L

**Table t7-jetem-5-2-s1:** 

*Lactic Acid*	4.1 mmol/L

**Table t8-jetem-5-2-s1:** 

*Creatine kinase*	*458 U/L*

**Table t9-jetem-5-2-s1:** 

*Venous blood gas*	
pH	7.25
pCO_2_	30 mmHg
pO_2_	60 mmHg
HCO_3_	12 mEq/L
O_2_ Saturation	98% on room air

**Table t10-jetem-5-2-s1:** 

*Urinalysis (UA)*	
Leukocyte esterase	negative
Nitrites	negative
Blood	none
Ketones	2+
Glucose	none
Color	dark yellow
White blood cells (WBC)	0–5 WBCs/high powered field (HPF)
Red blood cells (RBC)	5–10 RBCs/HPF
Squamous epithelial cells	0–5 cells/HPF
Specific gravity	1.015

**Figure f1-jetem-5-2-s1:**
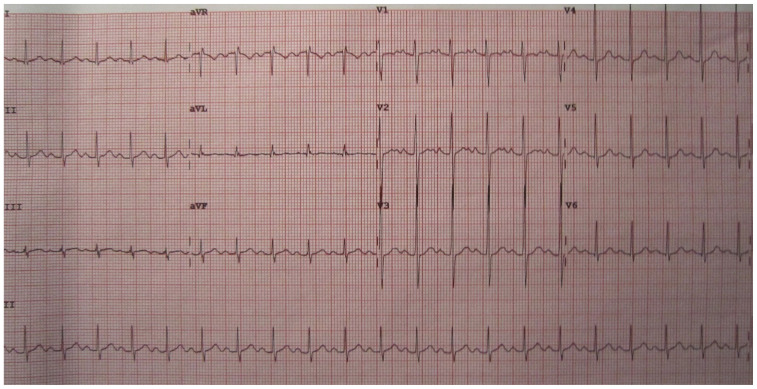


**Figure f2-jetem-5-2-s1:**
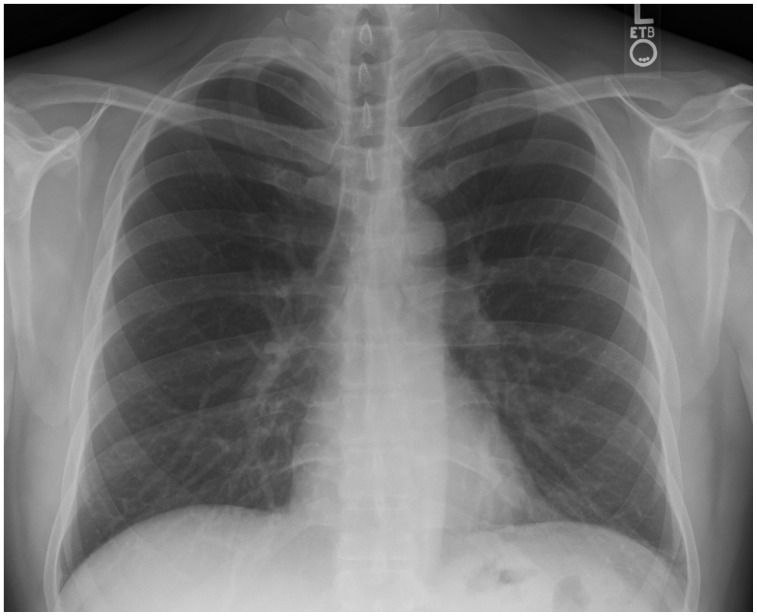


**Figure f3-jetem-5-2-s1:**
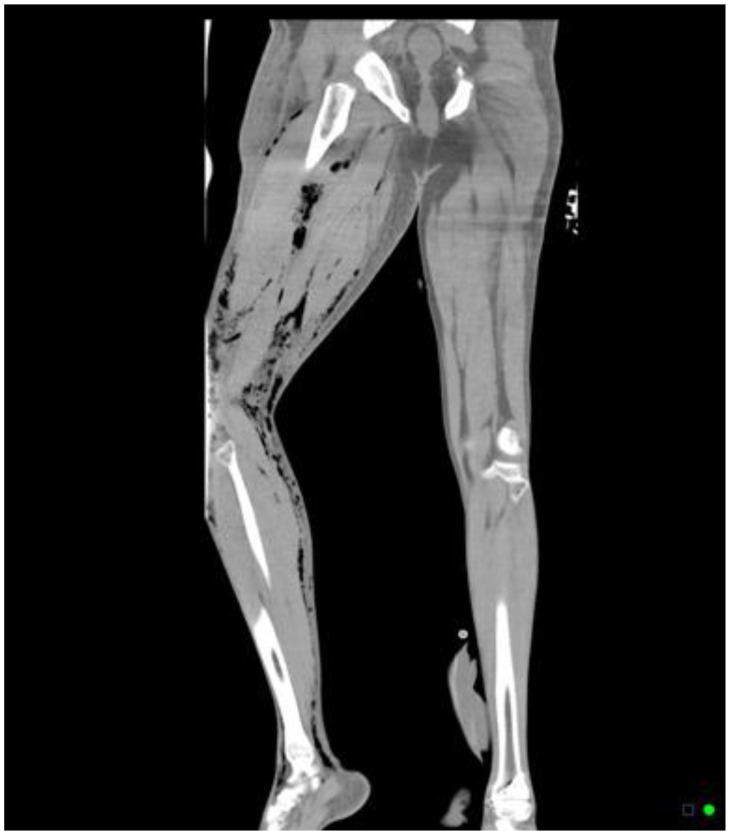


**Figure f4-jetem-5-2-s1:**
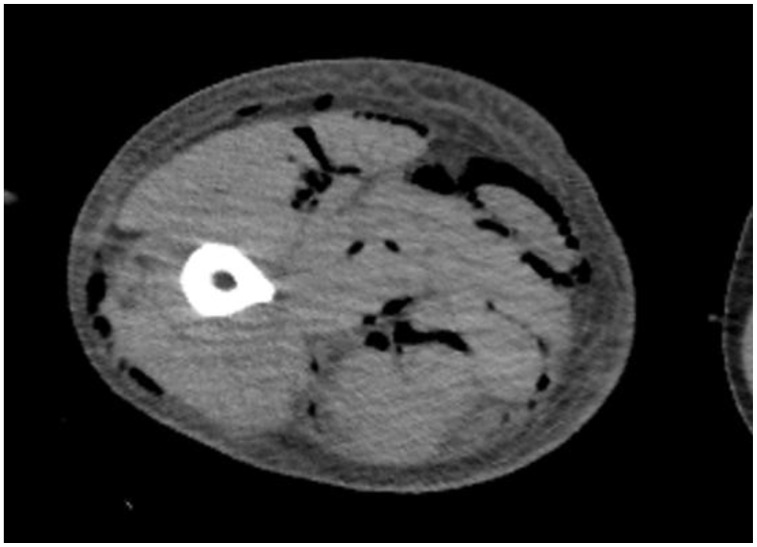


**Figure f5-jetem-5-2-s1:**
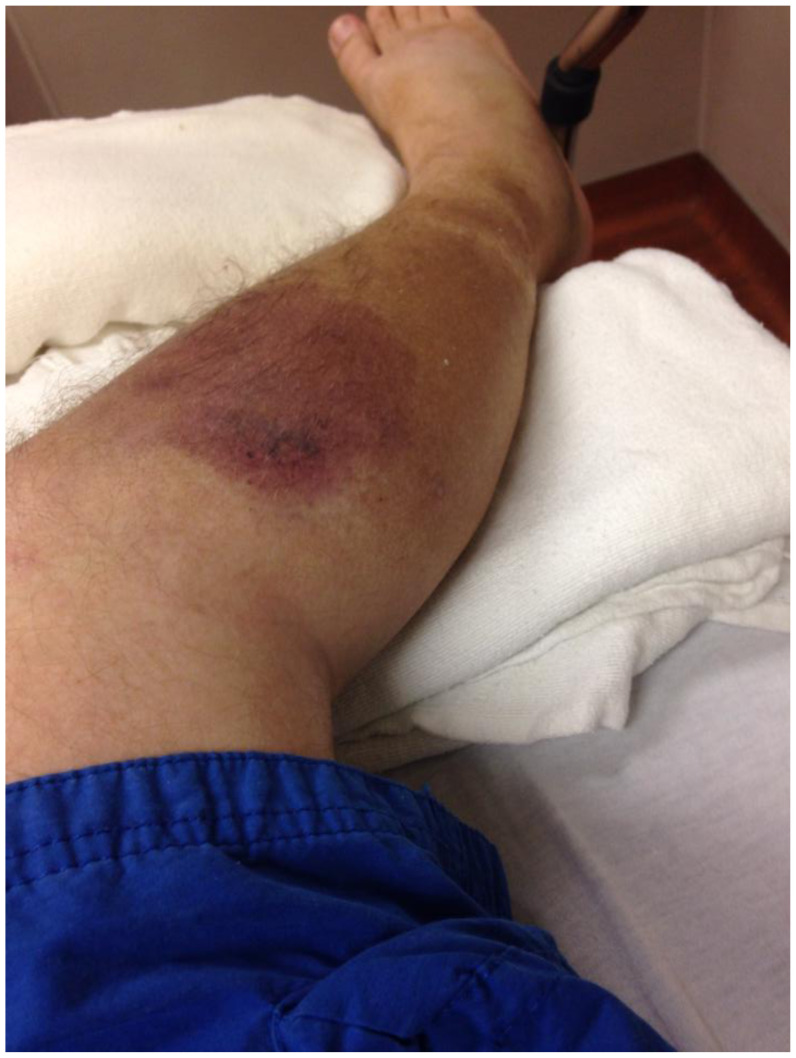


**Table t11-jetem-5-2-s1:** SIMULATION EVENTS TABLE:

Minute (state)	Participant action/ trigger	Patient status (simulator response) & operator prompts	Monitor display (vital signs)
0:00 (Baseline)	Case begins. Patient is placed on cardiac monitoring and participants should begin to obtain history.	Participants should begin by placing the patient on a monitor, obtaining a history and initiating a physical exam. Patient will be complaining of pain to his leg	No vitals should be displayed until requested T 100.3F HR 126 BP 110/68 RR 26 O2 98% RA
04:00	Completion of history and physical exam. IV fluids should be ordered.	If participants do not visually inspect the painful area, the patient should complain of increased pain and ask if they see anything there. Labs and imaging should result approximately two minutes after ordering these tests.	After 30cc/kg IV fluid bolus: T 100.3F HR 115 BP 120/78 RR 20 O2 98% RA Without 30cc/kg IV fluid bolus: T 102.3F HR 140 BP 98/60 RR 30 O2 98% RA
07:00	Team should voice concern for NF, initiate antibiotics and consult surgery.	Surgery consultant should request imaging be performed before they examine the patient. Participants must then clearly voice their concern for necrotizing fasciitis and need for STAT surgical consult before they agree to see the patient prior to imaging results. If the participants do not clearly relay their concerns, surgery will tell them they’ll call them back later and will hang up. If the team does not voice concern for necrotizing fasciitis or they do not consult surgery by minute 08:00, go to B vitals. If the team is not insistent on a STAT surgical consultant OR if antibiotics are not given by 08:00, patient will become more hypotensive at minute 08:00 (A vitals) with further decompensation at minute 10:00 (B vitals) until these are performed. If team requests a STAT surgical consult and orders empiric antibiotics by 08:00, vitals will remain unchanged from previous step (C or D vitals, depending on IV fluid bolus administered).	A: Without STAT surgical consult AND broadspectrum antibiotics by minute 08:00: T 102.3F HR 150 BP 80/50 RR 30 O2 98% RA B: Without STAT surgical consult AND broadspectrum antibiotics by minute 08:00: T 102.3F HR 160 BP 70/40 RR 35 O2 98% RA C: After STAT surgical consult and broadspectrum antibiotics with 30 cc/kg IVF bolus: T 100.3F HR 115 BP 120/78 RR 20 O2 98% RA D: After STAT surgical consult and broadspectrum antibiotics but without 30cc/kg IV fluid bolus: T 102.3F HR 140 BP 98/60 RR 30 O2 98% RA

**Table t12-jetem-5-2-s1:** 

**Critical Actions:** Place patient on continuous cardiac monitoring and obtain a full set of vitals Visualize the affected area by removing overlying clothing Initiate fluid resuscitation (30 cc/kg bolus) Initiate broad-spectrum antibiotics, including clindamycin Consult surgery for prompt surgical evaluation Admit patient to the ICU after OR management	0:00

**Table t13-jetem-5-2-s1:** 

	Milestone	Did not achieve level 1	Level 1	Level 2	Level 3
1	**Emergency Stabilization (PC1)**	□ Did not achieve Level 1	□ Recognizes abnormal vital signs	□ Recognizes an unstable patient, requiring intervention Performs primary assessment Discerns data to formulate a diagnostic impression/plan	□ Manages and prioritizes critical actions in a critically ill patient Reassesses after implementing a stabilizing intervention
2	**Performance of focused history and physical (PC2)**	□ Did not achieve Level 1	□ Performs a reliable, comprehensive history and physical exam	□ Performs and communicates a focused history and physical exam based on chief complaint and urgent issues	□ Prioritizes essential components of history and physical exam given dynamic circumstances
3	**Diagnostic studies (PC3)**	□ Did not achieve Level 1	□ Determines the necessity of diagnostic studies	□ Orders appropriate diagnostic studies. Performs appropriate bedside diagnostic studies/procedures	□ Prioritizes essential testing Interprets results of diagnostic studies Reviews risks, benefits, contraindications, and alternatives to a diagnostic study or procedure
4	**Diagnosis (PC4)**	□ Did not achieve Level 1	□ Considers a list of potential diagnoses	□ Considers an appropriate list of potential diagnosis May or may not make correct diagnosis	□ Makes the appropriate diagnosis Considers other potential diagnoses, avoiding premature closure
5	**Pharmacotherapy (PC5)**	□ Did not achieve Level 1	□ Asks patient for drug allergies	□ Selects an medication for therapeutic intervention, consider potential adverse effects	□ Selects the most appropriate medication and understands mechanism of action, effect, and potential side effects Considers and recognizes drug-drug interactions
6	**Observation and reassessment (PC6)**	□ Did not achieve Level 1	□ Reevaluates patient at least one time during case	□ Reevaluates patient after most therapeutic interventions	□ Consistently evaluates the effectiveness of therapies at appropriate intervals
7	**Disposition (PC7)**	□ Did not achieve Level 1	□ Appropriately selects whether to admit or discharge the patient	□ Appropriately selects whether to admit or discharge Involves the expertise of some of the appropriate specialists	□ Educates the patient appropriately about their disposition Assigns patient to an appropriate level of care (ICU/Tele/Floor) Involves expertise of all appropriate specialists
9	**General Approach to Procedures (PC9)**	□ Did not achieve Level 1	□ Identifies pertinent anatomy and physiology for a procedure Uses appropriate Universal Precautions	□ Obtains informed consent Knows indications, contraindications, anatomic landmarks, equipment, anesthetic and procedural technique, and potential complications for common ED procedures	□ Determines a back-up strategy if initial attempts are unsuccessful Correctly interprets results of diagnostic procedure

## INSTRUCTOR MATERIALS

**Case Title:** Necrotizing Fasciitis

**Case Description & Diagnosis (short synopsis):** A 34-year-old male with a history of type II diabetes presents with right posterior leg pain for two days.

Pain initially started after participating in a 5K race. Participants should perform a thorough physical exam to discover right posterior thigh erythema. Participants should also order intravenous fluids and start empiric antibiotics. Team should obtain imaging but not let testing delay surgical consultation. Patient should ultimately be admitted to the intensive care unit (ICU) after definitive management in the operating room (OR).

**Equipment or Props Needed:**

High-fidelity simulation mannequin

Facial diaphoresis may be accomplished with water applied with a spray bottle

Mannequin should be fully dressed, wearing pants that can be easily removed

Right posterior thigh erythema moulage (as demonstrated in the stimulus inventory for moulage purposes)

Angiocaths for peripheral intravenous access = 18g, 20g, 22g

Cardiac monitor

Pulse oximetry

Intravenous (IV) pole

Normal saline or lactated ringers (1L x2)

Simulated medications with labeling: vancomycin, Zosyn^®^, and clindamycin

**Confederates needed:**

Primary nurse. Faculty running the simulation may call in as surgical team, radiologist, and the intensivist.

**Stimulus Inventory:**

[Table t2-jetem-5-2-s1]

**Background and brief information:** Patient presents from home to the ED for evaluation of leg pain.

**Initial presentation:** Patient is a 34-year-old male with history of type 2 diabetes mellitus who presents with right thigh pain for two days.

The pain initially started as cramps following a 5K race two days ago, and it has progressively gotten worse throughout the day. Patient is diaphoretic and in obvious pain.

**How the scenario unfolds:** Patient is a 34-year-old male who presents from home with right leg pain. Participants should expose the patient’s right leg. Bloodwork will reveal leukocytosis and elevated lactate. Participants should initiate IV fluids and broad-spectrum antibiotics, including vancomycin, Zosyn^®^ and clindamycin. Participants should have high suspicion for NF and consult surgery emergently. Participants should not allow imaging to delay surgical evaluation. If there is a delay in fluid resuscitation, empiric antibiotics, or STAT surgical consultation, the patient will become more hypotensive and tachycardic. If these actions are not all performed, the patient will continue to decompensate. Once all of these actions have been performed, tachycardia and hypotension will improve, but the patient will still require emergent operative debridement and subsequent ICU admission.

**Critical actions:**

1. Place patient on continuous cardiac monitoring
2. Visualize the affected area by removing overlying clothing
3. Initiate fluid resuscitation (30 cc/kg bolus)
4. Initiate broad-spectrum antibiotics, including clindamycin
5. Consult surgery for prompt surgical evaluation
6. Admit patient to the ICU after OR management

**Case Title:** Necrotizing fasciitis

**Chief Complaint:** Patient is a 34-year-old male with right leg pain[Table t3-jetem-5-2-s1]

**General Appearance:** lying supine in bed, appears uncomfortable due to pain

**Primary Survey:**

- **Airway:** intact
- **Breathing:** clear to auscultation, tachypneic
- **Circulation:** regular tachycardic rhythm with 2+ symmetric pulses

**History:**

- **History of present illness:** 34-year-old male with a history of type II diabetes mellitus presents today with right leg pain for two days. He reports pain initially started following a 5K race. Pain is throbbing in nature, and progressively getting worse over the day. He denies any history of trauma to the site. He denies associated weakness, numbness or decreased sensation in the area. Denies IV drug use, allergies, recent new medications, new detergent or change of soap. Denies history of thromboembolism, recent travel, surgery, hemoptysis, chest pain, or dyspnea.
  Patient appears diaphoretic and uncomfortable secondary to pain.
- **Past medical history**: Diabetes mellitus type II, hypertension and obstructive sleep apnea
- **Past surgical history**: none
- **Medications**: metformin, lisinopril
- **Allergies**: none
- **Social history**: no alcohol use. Smokes 1 pack per day.
- **Family history**: noncontributory

**Secondary Survey/Physical Examination:**

- **General appearance:** 34-year-old male in obvious pain
- **HEENT:**
  ○ **Head:** diaphoretic
  ○ **Eyes:** within normal limits
  ○ **Ears:** within normal limits
  ○ **Nose:** within normal limits
  ○ **Throat:** within normal limits
- **Neck:** within normal limits
- **Heart:** regular tachycardic rhythm, otherwise, within normal limits
- **Lungs:** clear lung sounds bilaterally, mildly tachypneic at rest
- **Abdominal/GI:** within normal limits
- **Genitourinary:** within normal limits
- **Rectal:** within normal limits
- **Extremities:** Right posterior thigh has a 10-centimeter by 10-centimeter area of poorly demarcated erythema with surrounding tenderness to palpation. The tenderness is severe and extends 3cm beyond erythema in a circumferential pattern. No associated crepitance.
- **Back:** within normal limits
- **Neuro:** within normal limits
- **Skin:** as above, otherwise wnl
- **Lymph:** within normal limits
- **Psych:** within normal limits

**Results:**

[Table t4-jetem-5-2-s1]

[Table t5-jetem-5-2-s1]

[Table t6-jetem-5-2-s1]

[Table t7-jetem-5-2-s1]

[Table t8-jetem-5-2-s1]

[Table t9-jetem-5-2-s1]

[Table t10-jetem-5-2-s1]

*Electrocardiogram (EKG)*

Sinus Tachycardia

Heilman J. Sinus tachycardia as seen on ECG. In: Wikimedia Commons.

https://commons.wikimedia.org/wiki/File:Sinustachy.JPG. Published 15 June 2012. CC BY-SA 3.0.[Fig f1-jetem-5-2-s1]

*Portable Chest Radiograph (CXR)*

Stillwaterising. Chest X-ray PA. In: Wikimedia Commons.

https://commons.wikimedia.org/wiki/File:Chest_Xray_PA_3-8-2010.png. Published March 8, 2010. Public domain.[Fig f2-jetem-5-2-s1]

*Computed tomography (CT) right lower extremity*

O'Donnell C. Necrotizing fasciitis. In: Radiopaedia.org.

https://radiopaedia.org/cases/necrotizing-fasciitis-1?lang=us. Published February 29th 2012. CC BY-NC-SA 3.0.[Fig f3-jetem-5-2-s1]

*Computed tomography (CT) right lower extremity*

O'Donnell C. Necrotizing fasciitis. In: Radiopaedia.org.

https://radiopaedia.org/cases/necrotizing-fasciitis-1?lang=us. Published February 29th 2012. CC BY-NC-SA 3.0.[Fig f4-jetem-5-2-s1]

*External skin findings*

Doetsch. The start of necrotizing fasciitis in Joe Doetsch. In: Wikimedia Commons.

https://commons.wikimedia.org/wiki/File:Beginning_of_Necrotizing_Fasciitis_01.jpg. Published January 1, 2014. CC BY-SA 4.0.[Fig f5-jetem-5-2-s1]

## OPERATOR MATERIALS

[Table t11-jetem-5-2-s1]

### Diagnosis

Necrotizing Fasciitis

### Disposition

Admit to the ICU after OR management

## DEBRIEFING AND EVALUATION PEARLS

### Necrotizing Fasciitis

#### Pearls

- NF can be due to mono or polymicrobial infections, including gram positive organisms (GAS, Staph A, MRSA), gram-negative bacilli, and spore- or toxin-producing anaerobes. Clinical presentation may range from subtle deep tissue disease to fulminant presentation with sepsis or multi-organ failure.
- The most frequently reported presenting signs and physical exam findings are fever, erythema, swelling, pain out of proportion, and/or pain beyond visualized margin of infection.
- Differential diagnoses may include deep vein thrombosis, cellulitis, hematoma, clostridial myonecrosis, pyomyositis, and pyoderma gangrenosum.
- Risk factors for necrotizing fasciitis include immunosuppressive conditions (diabetes, cirrhosis, HIV), malignancy, alcoholism, recent surgery or trauma.
- Diabetes mellitus is the most common predisposing factor associated with increased mortality, increased hospital length of stay, and limb amputation.
- Disease course includes an evolution of symptoms over hours or a couple of days. Symptom development may be initially subtle but progress rapidly.
- The widely known “hard” clinical signs of bullae, necrotic tissue, anesthesia, and subcutaneous emphysema are more suggestive of necrotizing infection, but these are rare and late findings of the disease. Absence of these signs is not sufficient to rule out necrotizing infection.
- Prognosis is guarded, with reported mortality rates ranging from the 20s–40s percent.
- It is critical to have a high suspicion of index for necrotizing disease. A thorough history and physical exam are key in making an early clinical diagnosis.
- While the LRINEC score may predict morbidity and mortality, sensitivity is limited. Further studies are needed to support its widespread use, and a low score should not exclude cases from receiving expedited, aggressive care.
- The most commonly reported anatomical site for necrotizing infections is the lower extremity.
- Surgical evaluation for definitive surgical treatment is indicated if necrotizing infection is clinically suspected. Diagnostic imaging should not delay surgical evaluation.
- In addition to surgical intervention, early fluid resuscitation and IV antibiotics with broad spectrum coverage have been shown to significantly improve mortality.
- Choice of empiric antibiotics will depend on patient demographics as well as consideration of an immunocompromised status. Clindamycin is included as part of empiric therapy for its anti-toxin effects against streptococci and staphylococci strains.
- Appropriate disposition is to the OR with subsequent ICU level of care.

#### Other debriefing points

Closed-loop communication amongst team: was it used? Why or why not? Were there any implications of this during case execution?

Was the diagnosis difficult? What other diagnoses did you consider?

## SIMULATION ASSESSMENT

*Necrotizing Fasciitis*

Learner: _________________________________________

***Assessment Timeline***

This timeline is to help observers assess their learners. It allows observer to make notes on when learners performed various tasks, which can help guide debriefing discussion.

[Table t12-jetem-5-2-s1]

**Critical Actions:**

□ Place patient on continuous cardiac monitoring and obtain a full set of vitals
□ Visualize the affected area by removing overlying clothing
□ Initiate fluid resuscitation (30 cc/kg bolus)
□ Initiate broad-spectrum antibiotics, including clindamycin
□ Consult surgery for prompt surgical evaluation
□ Admit patient to the ICU after OR management

**Summative and formative comments:**

**Milestones assessment:**

[Table t13-jetem-5-2-s1]
