# Supplementary material for: Necrotizing Fasciitis
Source: J Educ Teach Emerg Med. 2020 Apr 15;5(2):S1–S25. doi: 10.21980/J84M1D (PMC10332573; doi:10.21980/J84M1D)

## Slide 1
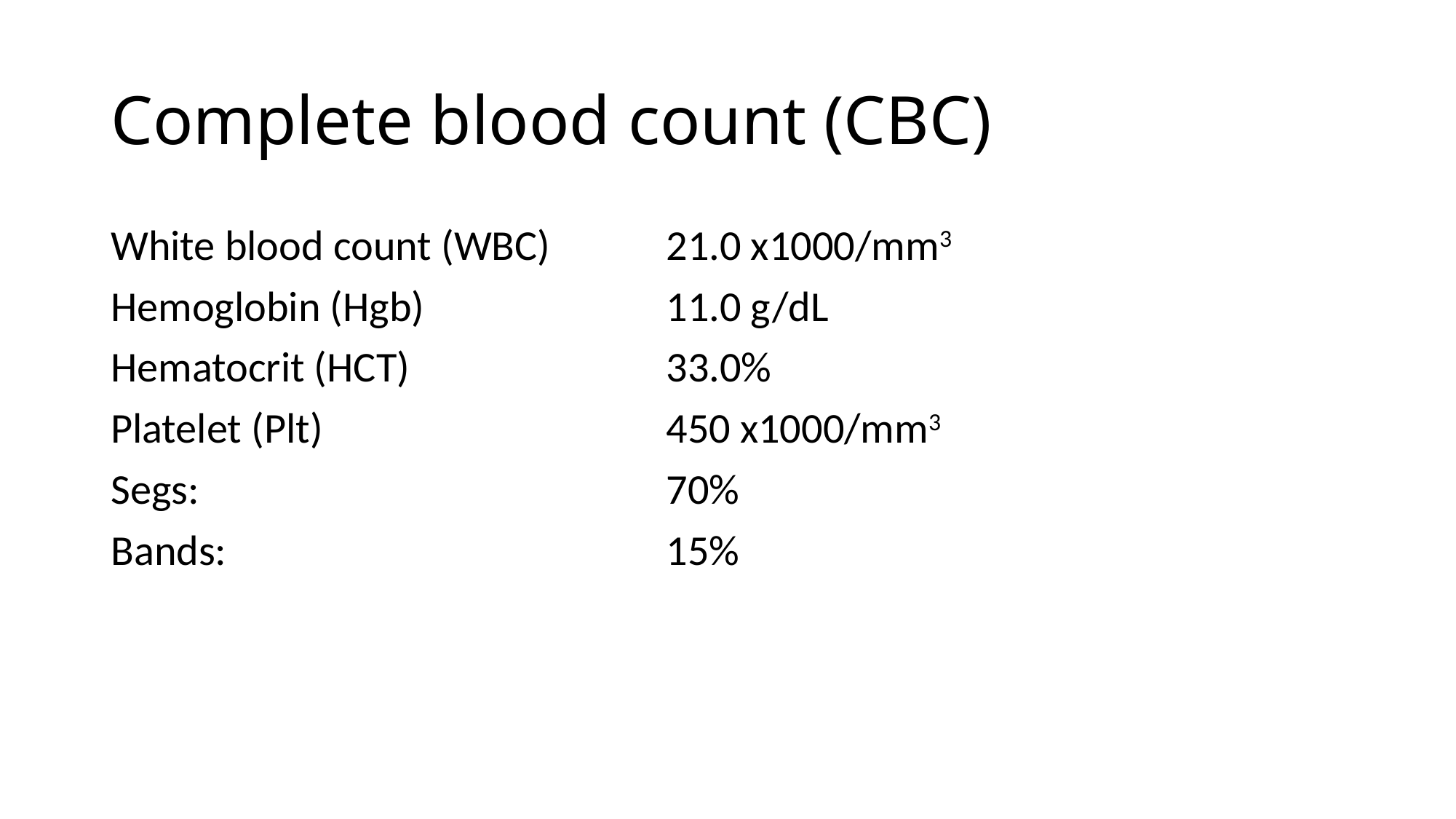

# Complete blood count (CBC)
White blood count (WBC) 	 21.0 x1000/mm3
Hemoglobin (Hgb) 		 11.0 g/dL
Hematocrit (HCT) 		 33.0%
Platelet (Plt) 			 450 x1000/mm3
Segs:					 70%
Bands:				 15%

## Slide 2
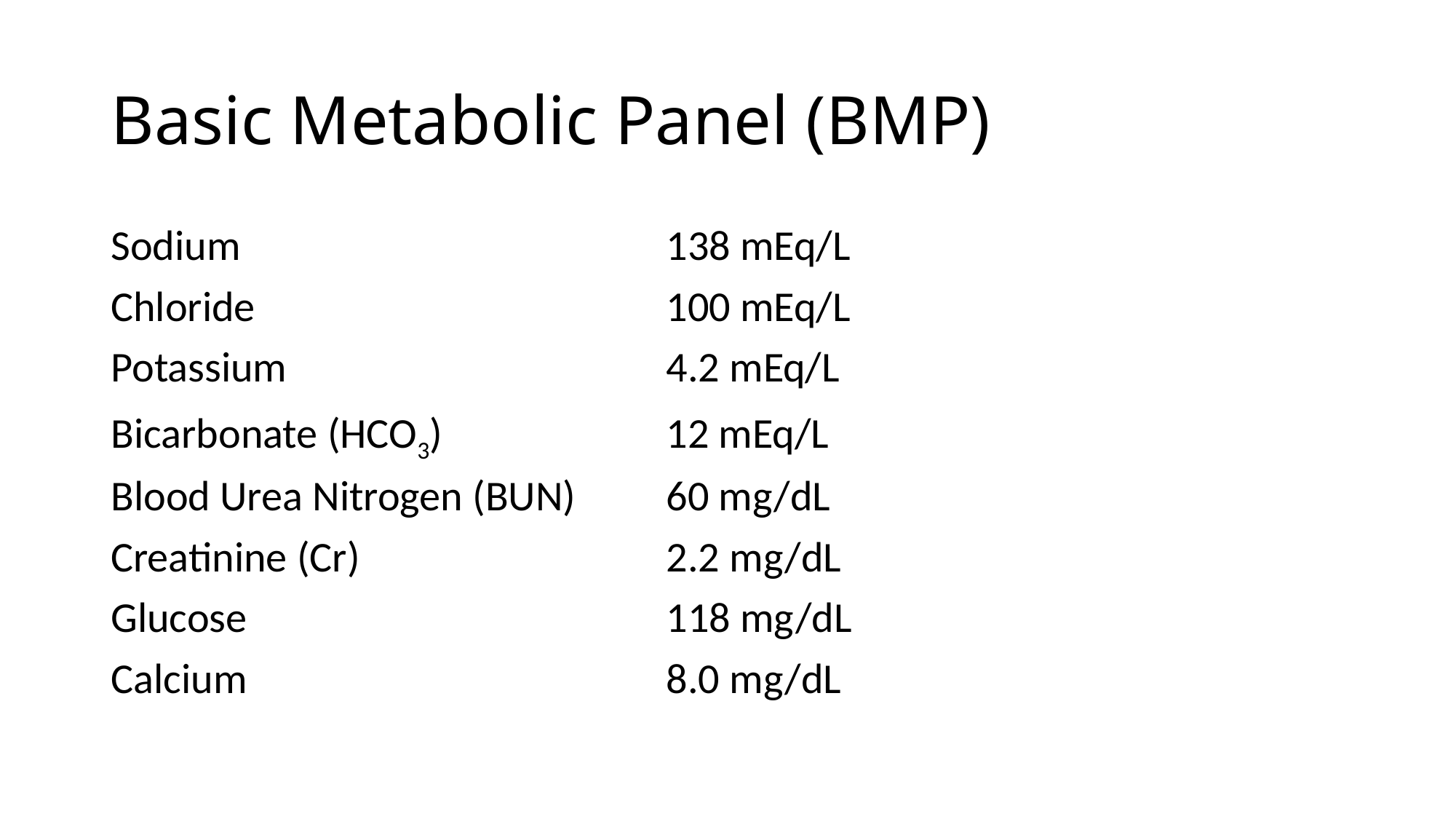

# Basic Metabolic Panel (BMP)
Sodium 				 138 mEq/L
Chloride 				 100 mEq/L
Potassium 				 4.2 mEq/L
Bicarbonate (HCO3) 		 12 mEq/L
Blood Urea Nitrogen (BUN) 	 60 mg/dL
Creatinine (Cr) 			 2.2 mg/dL
Glucose 				 118 mg/dL
Calcium				 8.0 mg/dL

## Slide 3
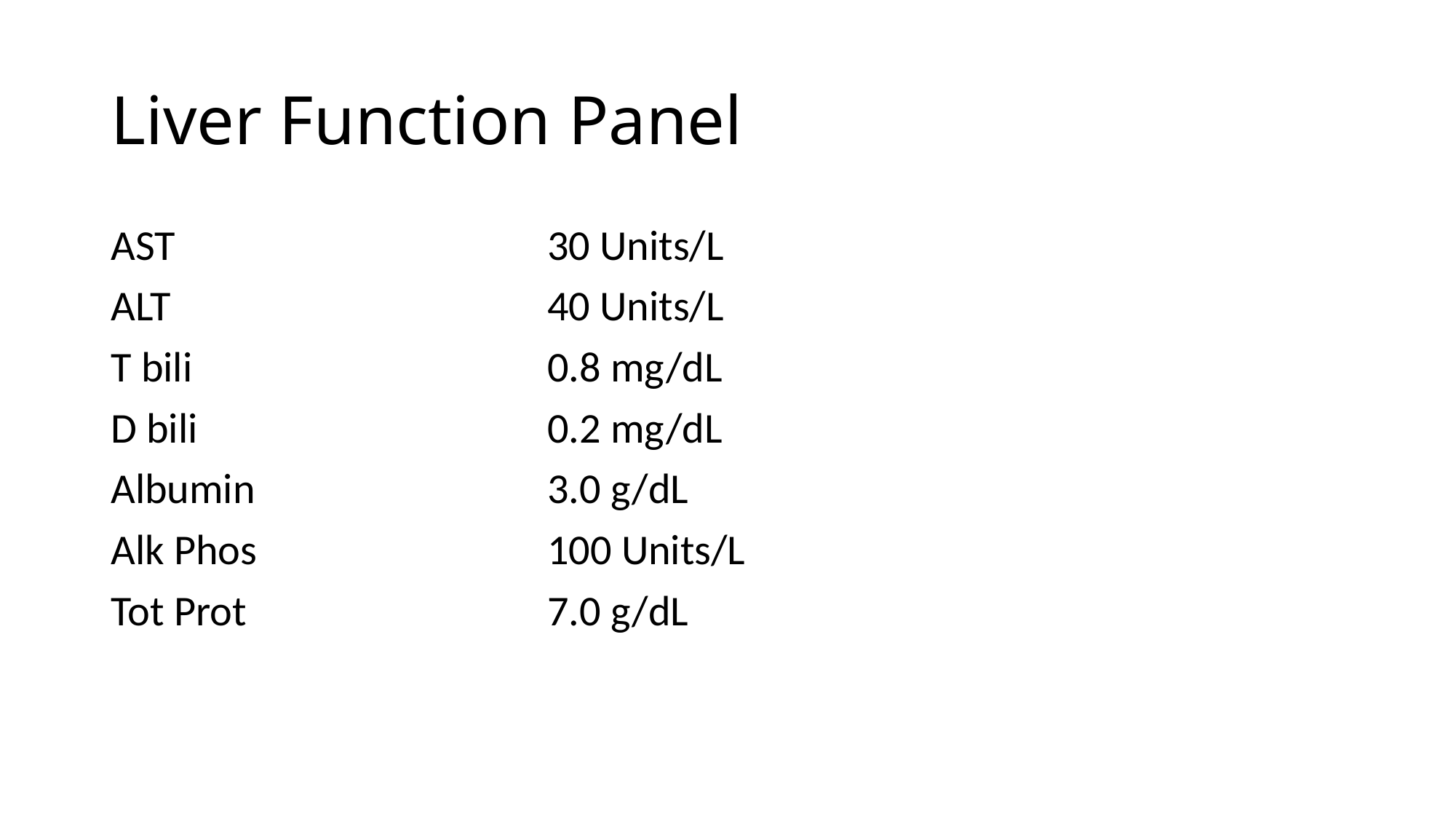

# Liver Function Panel
AST 				30 Units/L
ALT 				40 Units/L
T bili 				0.8 mg/dL
D bili 				0.2 mg/dL
Albumin 			3.0 g/dL
Alk Phos 			100 Units/L
Tot Prot 			7.0 g/dL

## Slide 4
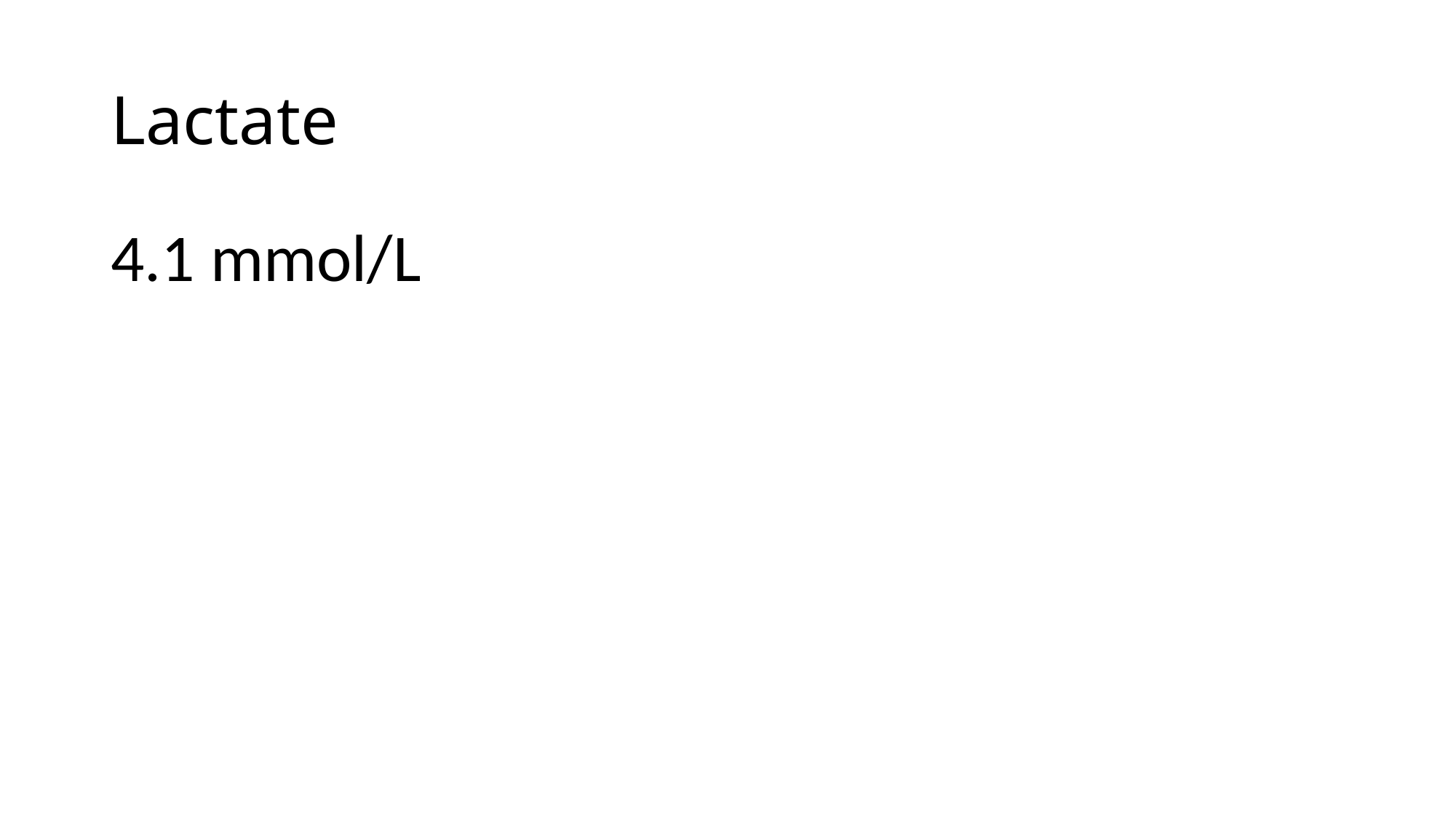

# Lactate
4.1 mmol/L

## Slide 5
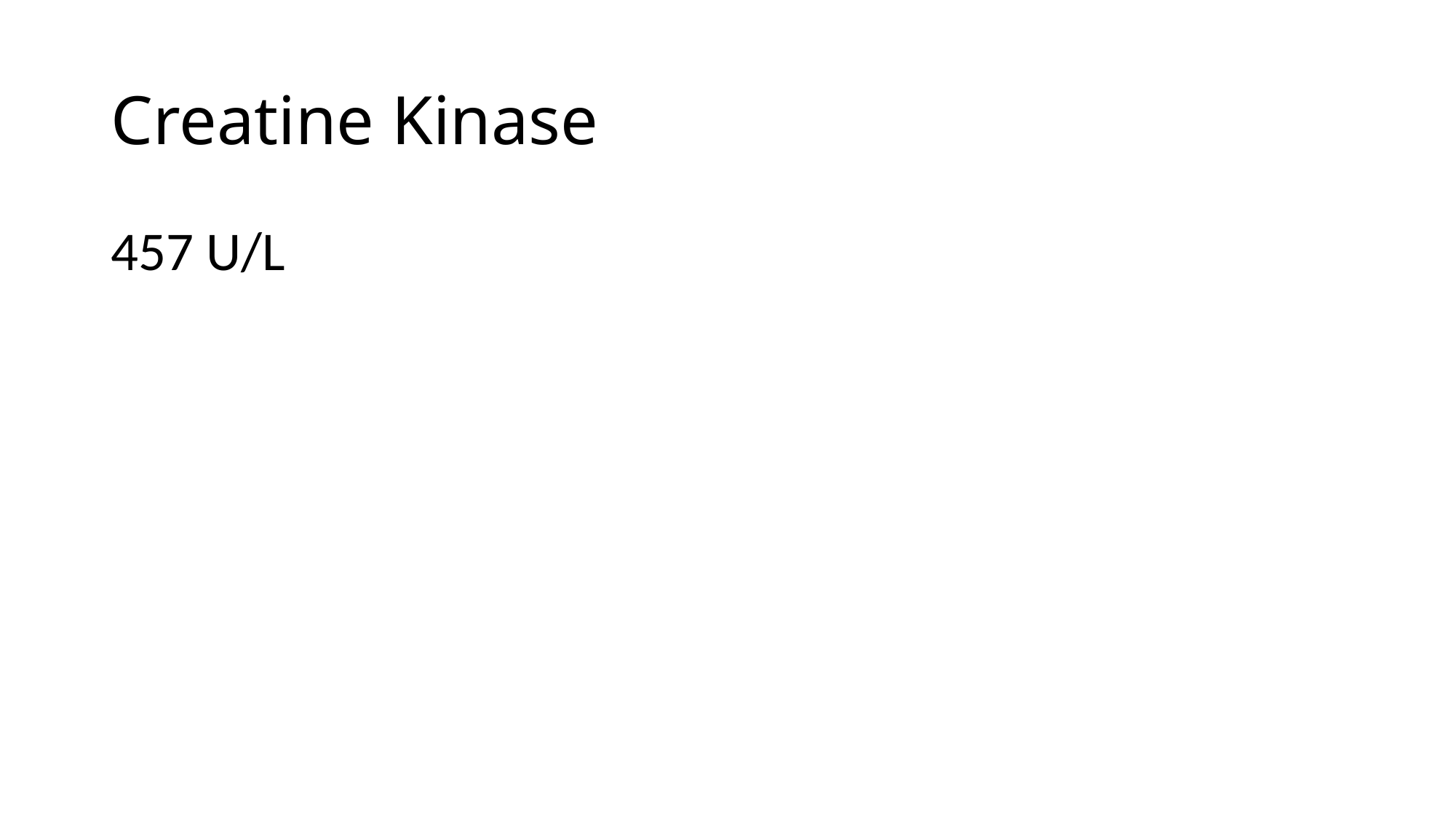

# Creatine Kinase
457 U/L

## Slide 6
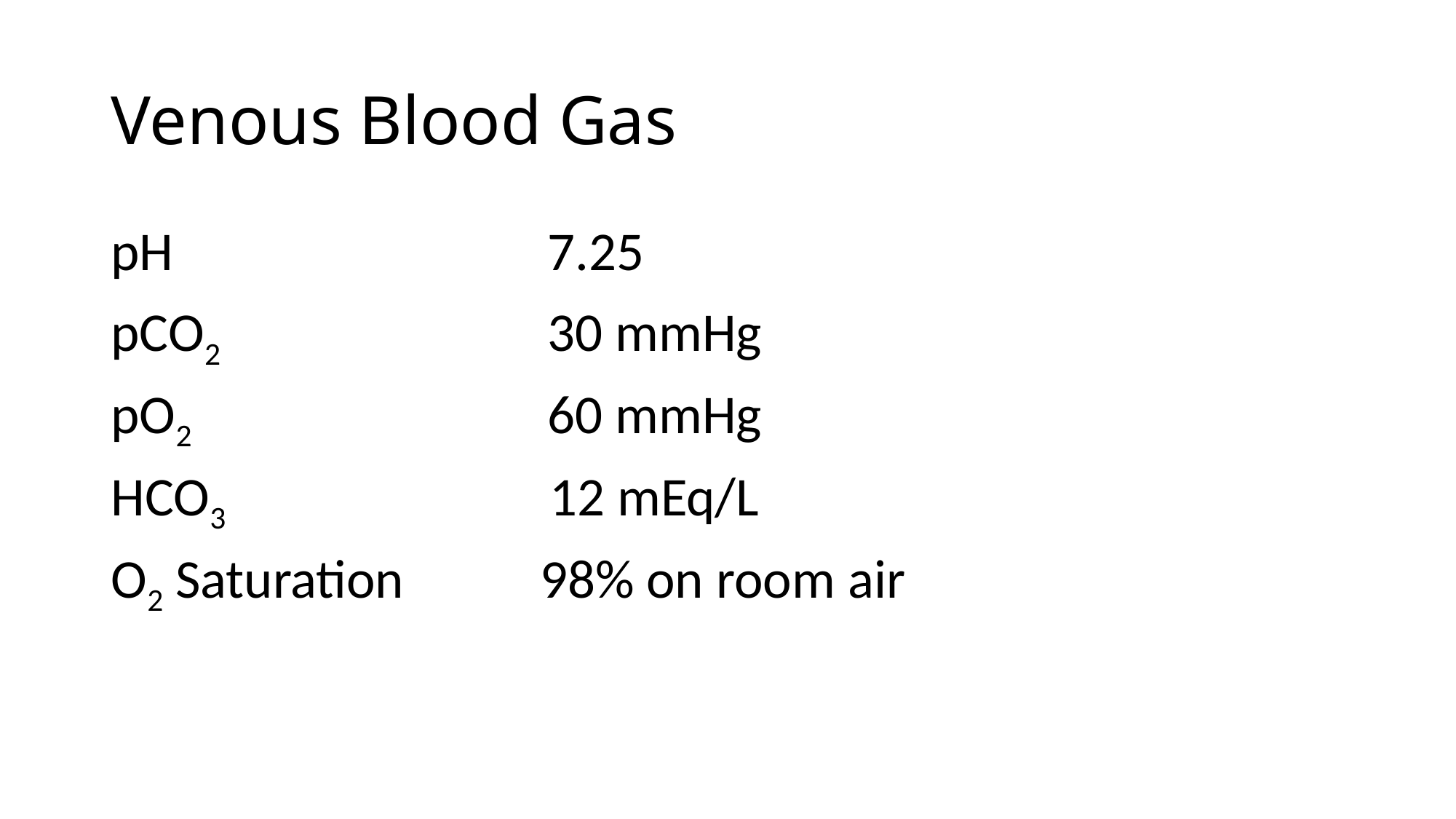

# Venous Blood Gas
pH 	 		7.25
pCO2 			30 mmHg
pO2 			60 mmHg
HCO3 		 12 mEq/L
O2 Saturation 98% on room air

## Slide 7
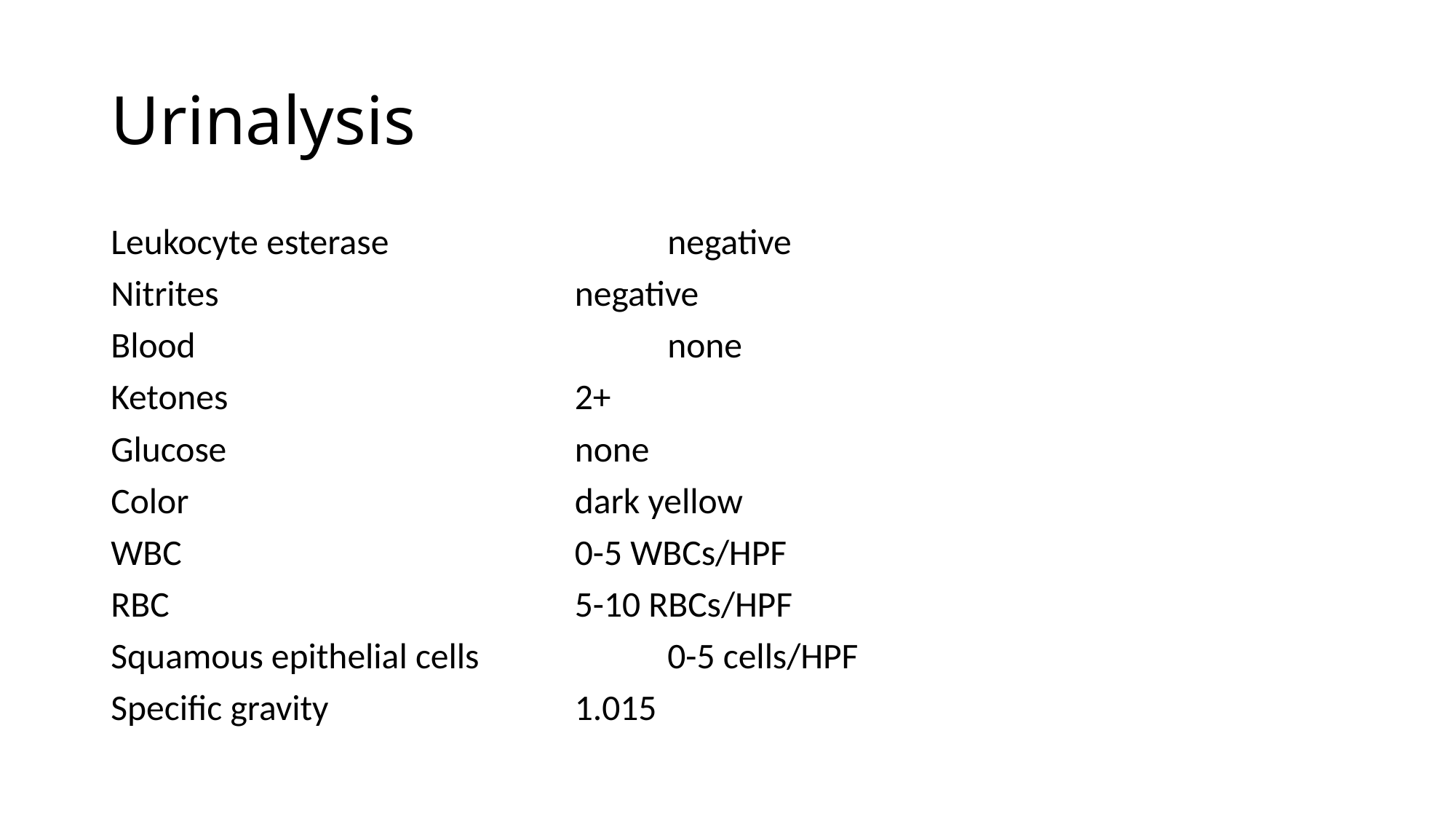

# Urinalysis
Leukocyte esterase 			negative
Nitrites				negative
Blood 					none
Ketones 				2+
Glucose 				none
Color 					dark yellow
WBC 					0-5 WBCs/HPF
RBC 					5-10 RBCs/HPF
Squamous epithelial cells 		0-5 cells/HPF
Specific gravity 			1.015

## Slide 8
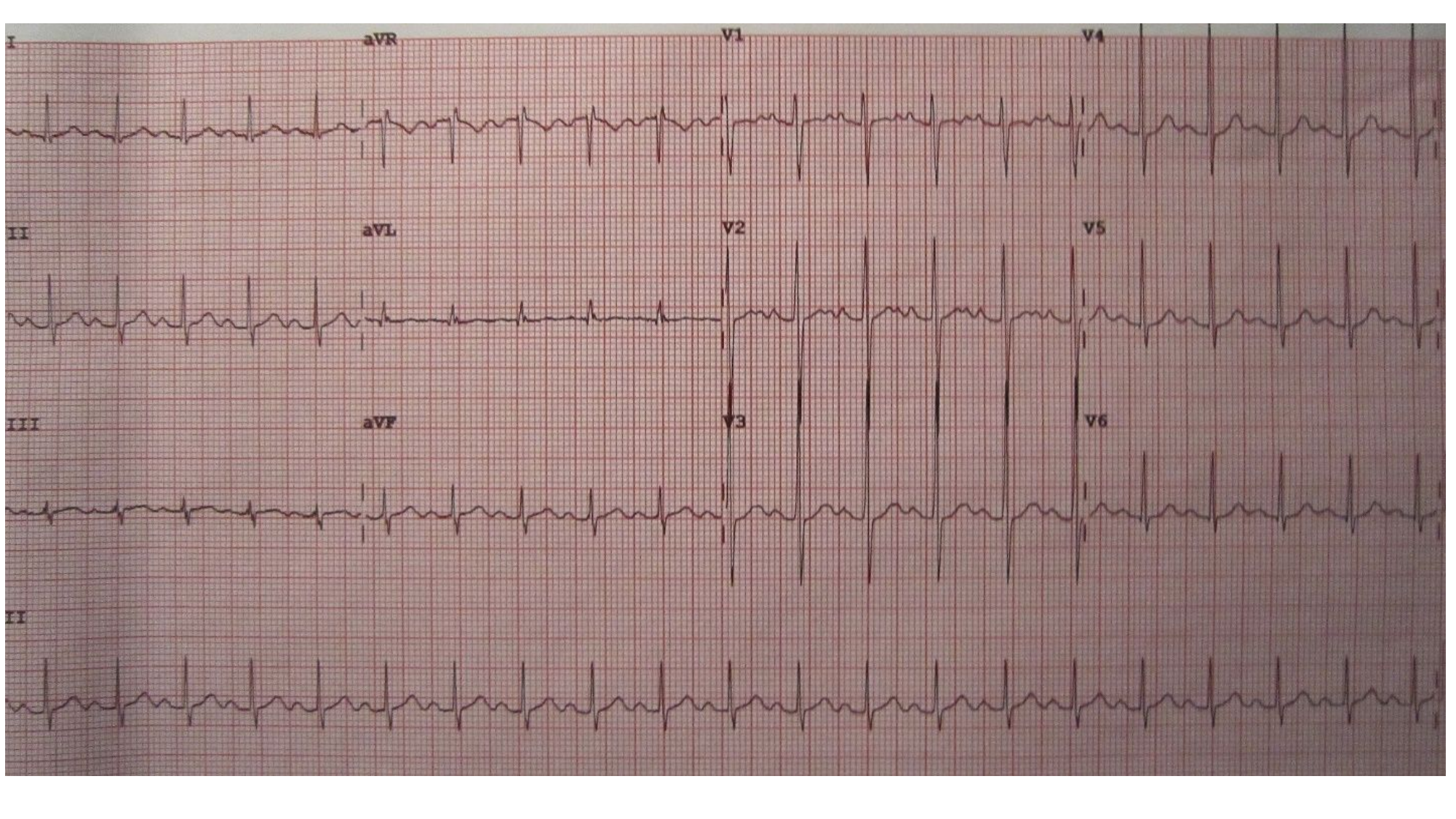

#

## Slide 9
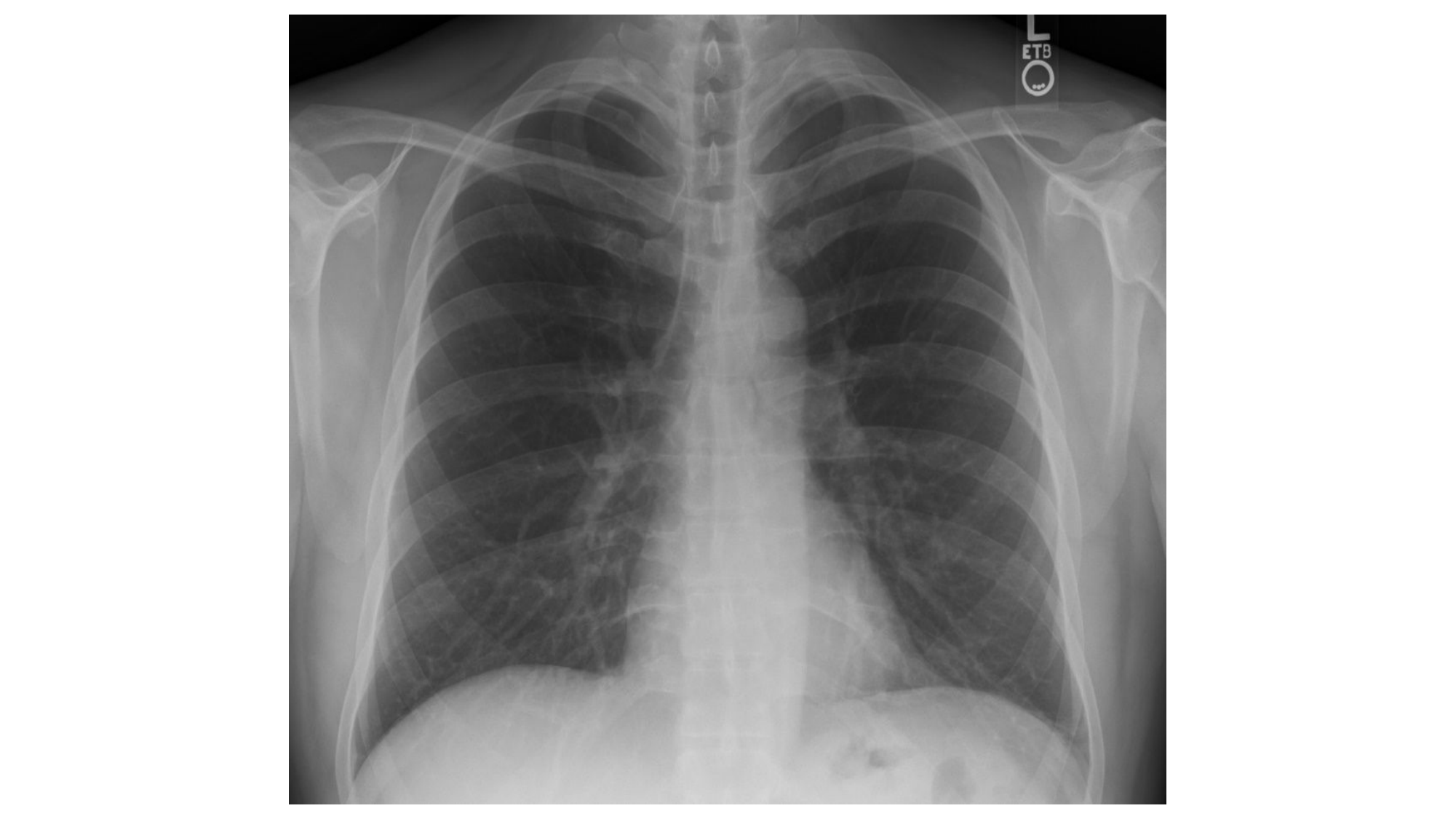

#

## Slide 10
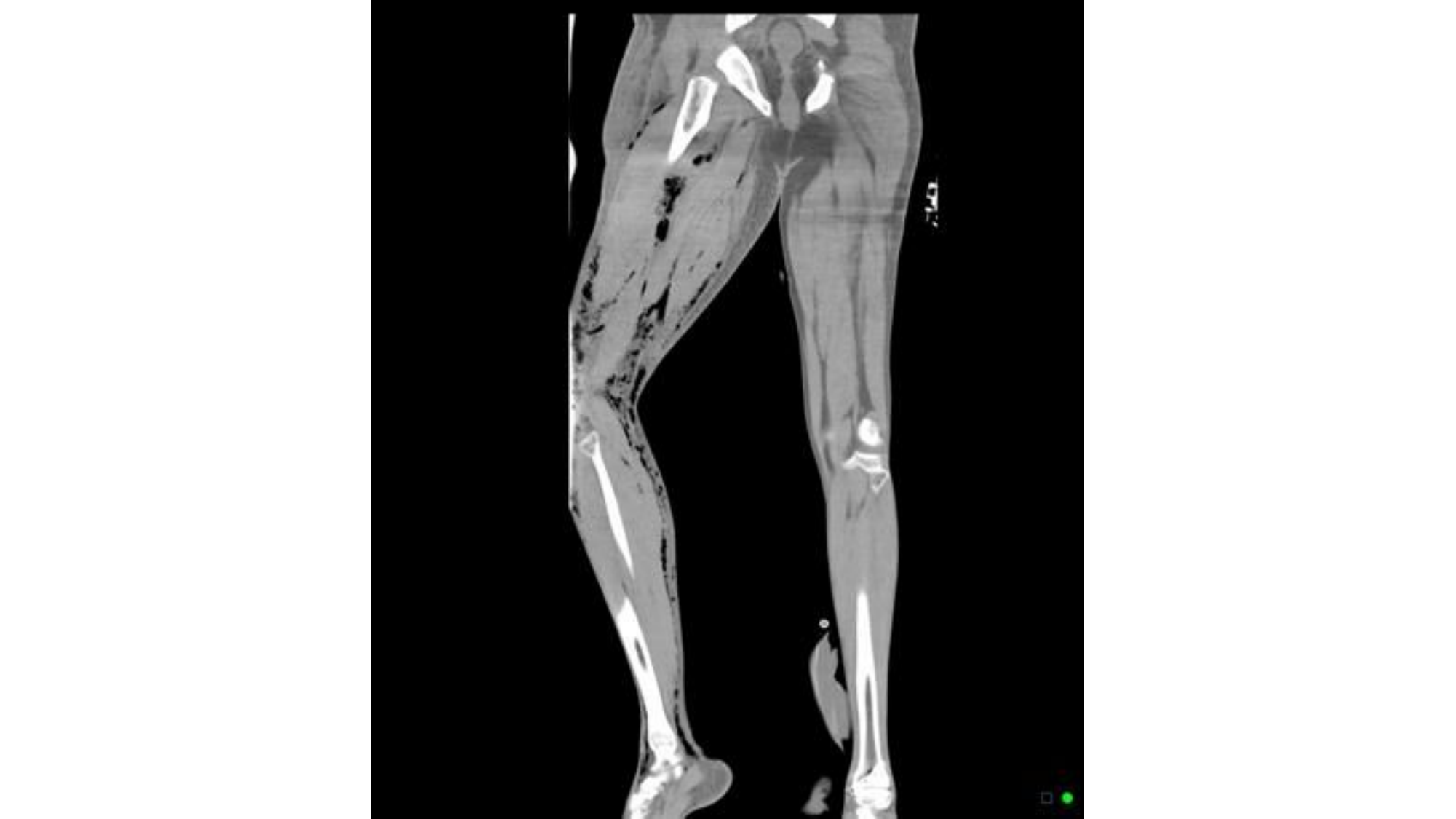

## Slide 11
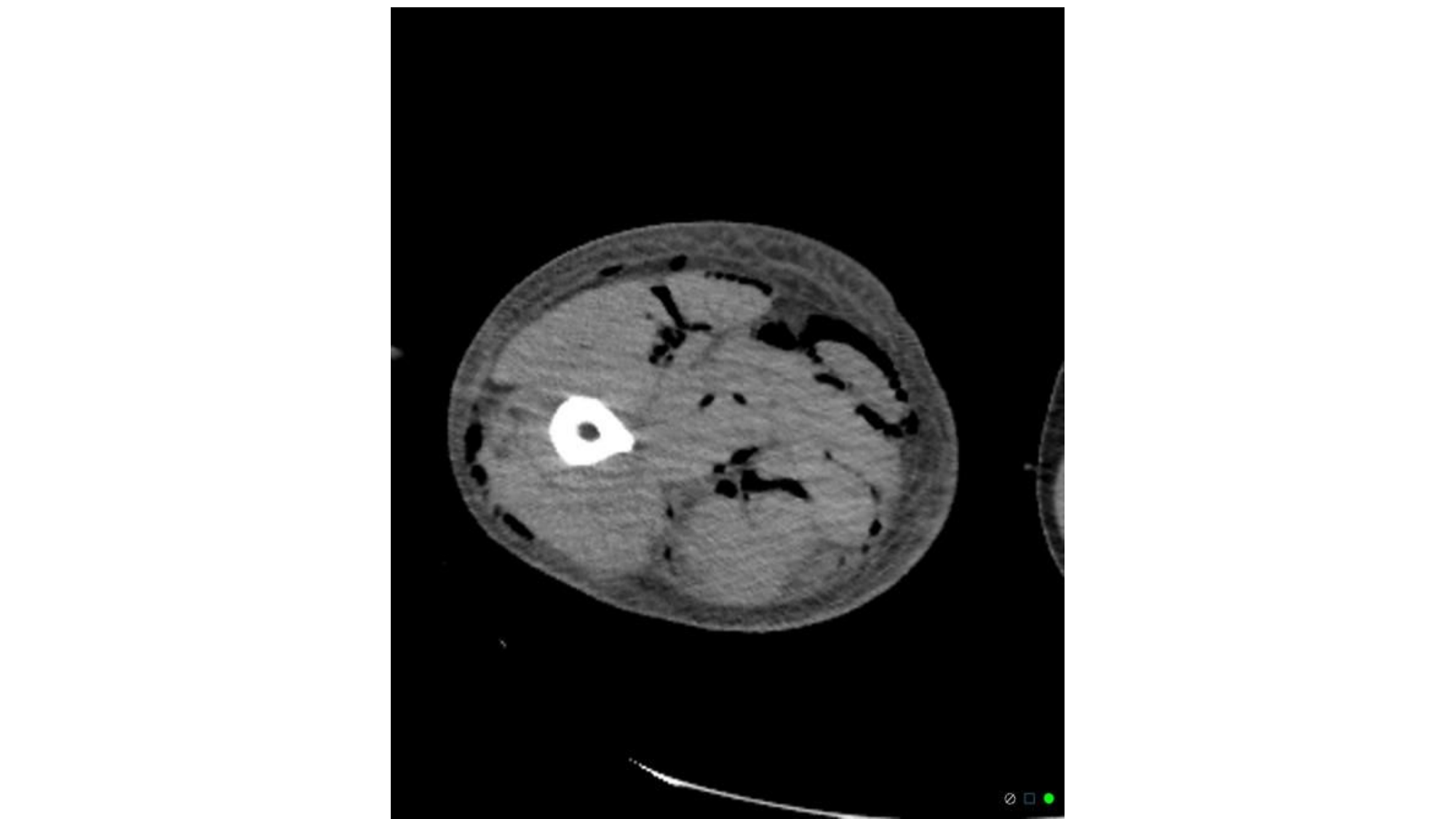

## Slide 12
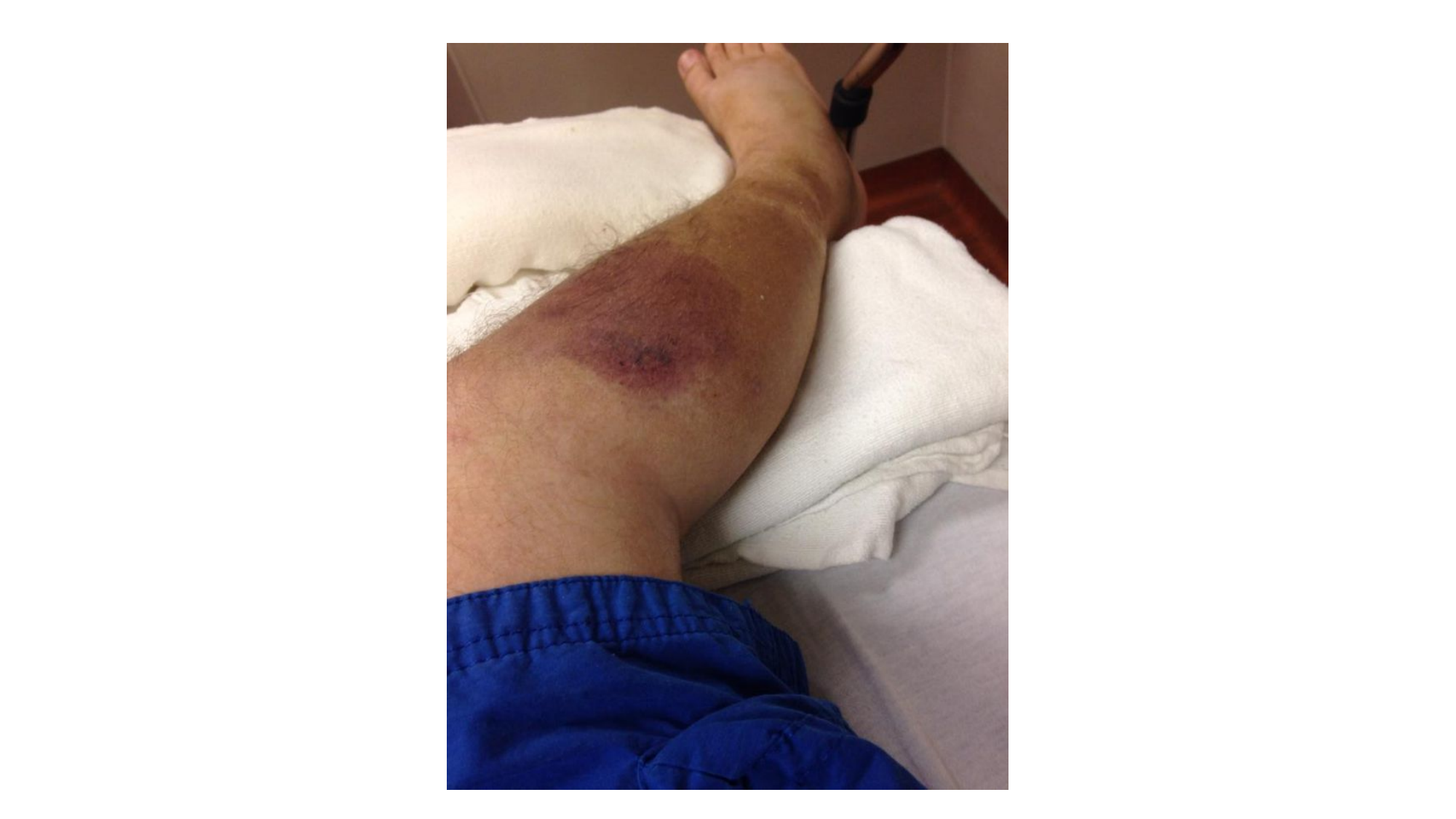

Supplement: Supplementary file 1 [file jetem-5-2-s1-supp1.pptx]
